# Comparative analysis of OCT-defined parapapillary beta and gamma zones between primary open angle glaucoma and primary angle closure glaucoma

**DOI:** 10.1038/s41598-022-15457-3

**Published:** 2022-06-30

**Authors:** Kunte Shang, Dongli Zhuang, Yi Dai

**Affiliations:** 1 Department of Ophthalmology & Visual Science, Eye & ENT Hospital, Shanghai Medical College, Fudan University, 83 Fenyang Road, Shanghai, 200031 China; 2 NHC Key Laboratory of Myopia (Fudan University), Key Laboratory of Myopia, Chinese Academy of Medical Sciences, and Shanghai Key Laboratory of Visual Impairment and Restoration (Fudan University), Shanghai, 200031 China

**Keywords:** Diagnostic markers, Glaucoma

## Abstract

The ophthalmoscopic beta zone of parapapillary atrophy has recently been proposed to divide into a gamma zone and a (new) beta zone based on OCT imaging. The present study was undertaken to compare the microstructural characteristics of parapapillary gamma and beta zones and their influencing factors between primary open angle glaucoma (POAG) and primary angle closure glaucoma (PACG). Seventy-three PACG patients that had no evidence of an acute attack and 78 POAG patients were enrolled. Patients were matched by propensity scores for age and visual field mean defect (MD) value. The area and angular extent of both zones were measured. In multivariate analysis, a larger beta zone was correlated with older age, severe MD value and longer axial length. A larger gamma zone was correlated with longer axial length. Older age and severe MD value were correlated with the concentric shape of beta zone. Comparing the PACG and POAG groups that adjusted for age and MD value, gamma zone was larger and more prevalent in the POAG group, while beta zone showed no significant difference. Taken separately, MD value was associated with the area and shape of beta zone in the PACG group. Axial length was associated with the temporal shape of beta zone in the POAG group. These data indicated that OCT-defined parapapillary beta and gamma zones exhibited different characteristics in two types of glaucoma. Clinically, the size of parapapillary beta zone may serve as a better indicator of glaucoma severity in eyes with PACG than that in POAG.

## Introduction

The ophthalmoscopic beta zone of parapapillary atrophy (PPA) characterized by the loss of retinal pigment epithelium (RPE) has long been associated with glaucoma^1–3^. It has been recently proposed that the ophthalmoscopic beta zone could be further divided into 2 zones: gamma zone and (new) beta zone based on spectral domain optical coherence tomography (SD-OCT) imaging. Gamma zone was defined as the parapapillary region free of Bruch’s membrane and beta zone was defined as the parapapillary region with the continued presence of Bruch’s membrane and absence of RPE^4,5^. Several lines of evidence have suggested that OCT-defined parapapillary beta zone was in association with aging, glaucoma, and to a lower degree with myopia. While OCT-defined parapapillary gamma zone was strongly associated with axial myopia^5–7^.

The mechanism on the development of PPA in glaucoma has not yet understood. Moreover, the clinical value of PPA with regard to the diagnosis of primary open angle glaucoma (POAG) remains controversial^8–10^. In the ocular hypertension treatment study, there was no difference for the size of ophthalmoscopic beta zone between cases that developed POAG and controls in both baseline and follow-up^8^. Ehrlich and Radcliffe reported that adding the parameter of PPA to a model already containing commonly assessed variables including age, intraocular pressure, central cornea thickness, and cup/disc ratio, does not improve the ability to discriminate between POAG and normal eyes^9^. On the other hand, the morphological feature of OCT-defined parapapillary beta and gamma zones in eyes with different glaucoma type has only been scarcely addressed, if any. A previous study reported that the ophthalmoscopic beta zone size in chronic primary angle closure glaucoma (PACG) is much smaller than that in POAG, suggesting the PPA has a different relationship to the structural and functional optic disc changes in two types of glaucoma^11^. Therefore, the current study was undertaken to compare the microstructural characteristics of OCT-defined parapapillary gamma and beta zones and their influencing factors between eyes with POAG and PACG.

## Results

### Demographic characteristics

The study included 172 primary glaucoma patients. Among them, 12 patients were excluded for incomplete data, and 9 patients were excluded for poor image quality. 73 eyes from 73 PACG patients and 78 eyes from 78 POAG patients were finally analyzed. After propensity score matching, 60 pairs of 1:1 two-variable matched patients (age and MD value) were generated. Overall, the mean age was 55.43 ± 13.93 years (range, 20–80 years), the mean axial length was 23.84 ± 1.66 mm (range, 20.45–28.77 mm), and the mean MD value was − 14.52 ± 8.96 (range − 1.51 to − 32.03 dB). All eyes were phakic.

The demographic and clinical characteristics of patients in the PACG group and POAG group before and after matching are shown in Table 1. The two groups did not vary significantly in MD value and cpRNFL thickness. In comparison with the POAG group, mean axial length was significantly shorter (*P* < 0.001), mean area of gamma zone was significantly smaller (*P* < 0.001), mean IOP was significantly higher (*P* = 0.017), mean age was significantly older (*P* = 0.02), and mean area of beta zone was significantly larger (*P* = 0.031) in the PACG group. After propensity score matching for both age and MD value, mean area of beta zone no longer showed significant difference between the PACG and POAG groups (*P* = 0.493).

**Table 1:** Demographic and clinical characteristics of patients before and after PSM in PACG and POAG groups.

Variable	Before PSM		*P*-value	After PSM		*P*-value
	PACG (n = 73)	POAG (n = 78)		PACG (n = 60)	POAG (n = 60)	
Age, y	58.0 ± 10.85	52.97 ± 15.20	0.02	57.43 ± 12.80	55.80 ± 10.41	0.445
Male, n (%)	40 (54.79%)	51 (65.38%)	0.184	34 (56.67%)	37 (61.67%)	0.577
Spherical equivalent in diopters	0.93 ± 2.05	− 4.26 ± 3.95	< 0.001	1.12 ± 2.06	− 3.65 ± 3.66	< 0.001
Axial length, mm	22.68 ± 0.82	24.76 ± 1.58	< 0.001	22.60 ± 0.82	24.52 ± 1.47	< 0.001
MD value, dB	− 13.90 ± 8.95	− 15.09 ± 9.13	0.444	− 14.35 ± 9.03	− 12.83 ± 7.67	0.349
cpRNFL thickness	72.72 ± 32.16	62.87 ± 18.13	0.094	68.82 ± 31.91	65.97 ± 17.43	0.545
Beta zone area, mm^2^	0.82 ± 0.85	0.70 ± 0.51	0.287	0.70 ± 0.55	0.77 ± 0.53	0.493
Gamma zone area, mm^2^	0.10 ± 0.21	0.28 ± 0.35	< 0.001	0.08 ± 0.19	0.26 ± 0.36	0.001
Disc area, mm^2^	3.23 ± 0.02	2.53 ± 0.47	0.117	3.21 ± 0.02	2.60 ± 0.47	0.159
**Beta zone shape**			0.927*			0.488*
Concentric shape	22 (30.1%)	23 (29.5%)		15 (23.6%)	21 (27.3%)	
Temporal shape	44 (60.3%)	46 (59.0%)		39 (65.5%)	34 (60.0%)	
Non-beta zone	7 (9.6%)	9 (11.5%)		6 (10.9%)	5 (12.7%)	
IOP, mmHg	22.11 ± 10.11	17.89 ± 5.17	0.017	20.87 ± 9.44	17.58 ± 4.35	0.086

PSM: propensity score matching; PACG: primary angle-closure glaucoma; POAG: primary open angle glaucoma; MD: mean deviation; RNFL: retinal nerve fiber layer; IOP: intraocular pressure.

*Chi-square test was performed to compare the proportions of beta zone shape between POAG and PACG groups.

### Factors associated with parapapillary beta zone and gamma zone

**Figure 1:**
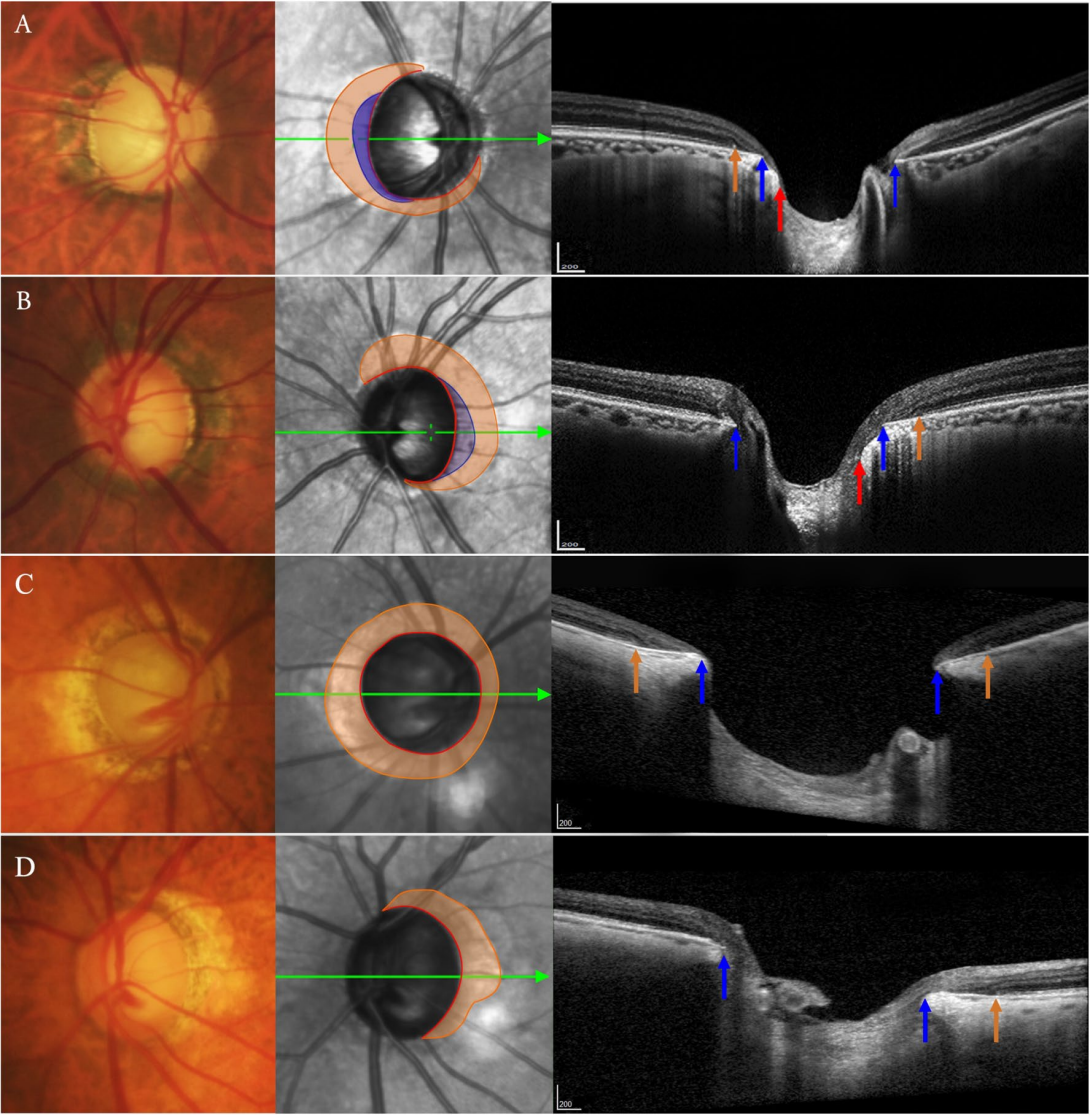
Illustration of measurement of parapapillary beta and gamma zones in bilateral eyes of glaucoma patients. (**A**,**B**) The size of parapapillary beta zone (orange shadow) and parapapillary gamma zone (blue shadow) was similar between the severe right eye and the moderate left eye in a POAG patient. (**C**,**D**) The severe right eye showed a concentric shape of parapapillary beta zone in a PACG patient, while the moderate left eye showed a relatively smaller parapapillary beta zone. Red arrow/line: optic disc margin; Blue arrow/line: the end of Bruch’s membrane; Orange arrow/line: the end of RPE.

**Figure 2:**
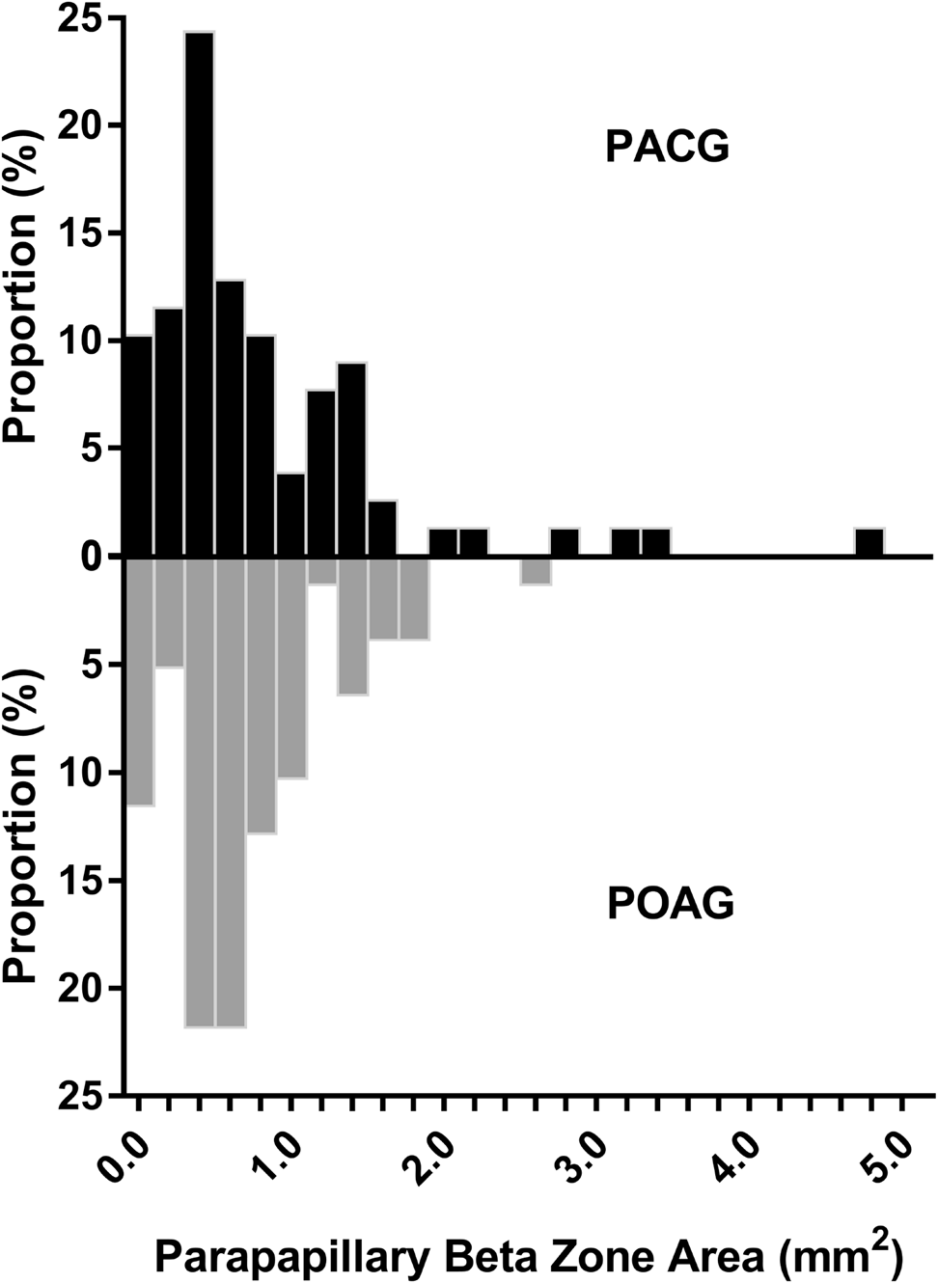
Distribution of the area of parapapillary beta zone in the POAG and PACG groups.

Among all patients included, parapapillary beta zone (mean area 0.76 ± 0.69 mm^2^, range 0.18–4.84 mm^2^) was present in 135 eyes, including 66 (90.4%) eyes in the PACG group and 69 (88.5%) eyes in the POAG group (Figs. 1, 2). Parapapillary gamma zone (mean area 0.19 ± 0.30 mm^2^, range 0.06–1.42 mm^2^) was present in 54 eyes, including 16 (21.9%) eyes in the PACG group and 38 (48.1%) eyes in the POAG group.

To investigate the associated factors with parapapillary beta zone, we first conducted the univariate and multivariate linear regression on beta zone area in the whole study group. In univariate analysis, a larger beta zone area was significantly associated with older age (*P* < 0.001), severe MD value (*P* = 0.041) and sex (*P* = 0.043). Model building for multivariate analysis began with the list of independent parameters including age, sex, axial length, IOP, MD, cpRNFL thickness and glaucoma type. Non-significant parameters were removed step by step from this full model. In final model, a larger beta zone area was associated with older age (*P* < 0.001), severe MD value (*P* = 0.002) and longer axial length (*P* = 0.029) (Table 2).

**Table 2:** Linear regression analysis of variables associated with the area of parapapillary beta zone in the whole study group.

Variable	Univariate analysis		Multivariate analysis	
	P-value	standardized coefficient beta	P-value	Standardized coefficient beta
Age, y	< 0.001	0.328	< 0.001	0.451
Sex	0.043	− 0.166	0.859	− 0.023
Axial length, mm	0.393	− 0.074	0.029	0.241
IOP, mmHg	0.208	− 0.134	0.085	− 0.219
Glaucoma type	0.287	− 0.088	0.153	− 0.182
MD value, dB	0.054	0.166	0.002	0.344
cpRNFL thickness	0.297	− 0.089	0.449	0.097

All the univariate variables in the table are included in the multivariate analysis.

MD: mean deviation; RNFL: retinal nerve fiber layer; IOP: intraocular pressure.

We then conducted the univariate and multivariate linear regression on beta zone area in the PACG group and POAG group, respectively. In multivariate model, beta zone area in the POAG group was finally associated with age (*P* = 0.007), while in the PACG group it was finally associated with age (*P* = 0.005) and MD value (*P* = 0.009) (Table 3). We further performed a paired sample t-test in eyes with bilateral POAG in 45 patients whose MD value differed by more than 2 dB. The results also showed that there was no significant difference on beta zone area between severe eyes and mild eyes with POAG (*P* = 0.576).

**Table 3:** Multivariate regression analysis of variables associated with the area of parapapillary beta zone in PACG and POAG groups.

Variable	PACG		POAG	
	P-value	Standardized coefficient beta	P-value	Standardized coefficient beta
Age, y	0.005	0.377	0.007	0.373
Sex	0.968	− 0.006	0.563	− 0.075
Axial length, mm	0.23	0.18	0.875	− 0.158
IOP, mmHg	0.188	− 0.215	0.71	− 0.049
MD value, dB	0.009	0.351	0.954	0.012
cpRNFL thickness	0.413	0.124	0.636	− 0.097

PACG: primary angle-closure glaucoma; POAG: primary open angle glaucoma; MD: mean deviation; RNFL: retinal nerve fiber layer; IOP: intraocular pressure.

Multivariate linear regression analyses were also performed to investigate factors related to parapapillary gamma zone area. In multivariate analysis model for gamma zone area, parameters including beta zone area (P = 0.099), MD value (P = 0.825), age (P = 0.817), IOP (P = 0.630), glaucoma type (P = 0.592), and mean cpRNFL thickness (P = 0.563). In final model, a larger gamma zone area was only associated with axial length (P < 0.001).

### Beta zone subtype according to angular extent

Among 135 glaucomatous eyes with parapapillary beta zone, mean angular extent was 196.0 ± 112.5° (range 60–360°). Among them, 90 eyes were classified into the temporal shape group and 45 eyes were in the concentric shape group. The proportions of different shape of parapapillary beta zone showed no significant difference between eyes with PACG and POAG (*P* > 0.05, Table 1). Compared with the concentric shape group, the temporal shape group showed a significant younger age (*P* < 0.001), smaller beta zone area (*P* < 0.001) and longer axial length (*P* = 0.039), while IOP, MD value, cpRNFL, gamma zone area showed no significant difference (*P* > 0.05). In multivariate logistic analysis of the whole group, the factors associated with concentric or temporal shape were age (OR = 1.081, *P* = 0.001) and MD value (OR = 1.064, *P* = 0.034) (Table 4).

**Table 4:** Logistic regression analysis of variables associated with concentric/temporal shape of parapapillary beta zone.

Variable	Univariate analysis		Multivariate analysis	
	P-value	OR (95%CI)	P-value	OR (95%CI)
Age, y	< 0.001	1.067 (1.031–1.104)	0.016	1.087 (1.016–1.162)
Sex	0.025	0.432 (0.207–0.901)	0.492	NA
Axial length, mm	0.043	0.750 (0.567–0.991)	0.275	NA
IOP, mmHg	0.053	0.934 (0.871–1.001)	0.052	NA
Glaucoma type	0.903	0.956 (0.467–1.957)	0.106	NA
MD value, dB	0.309	1.022 (0.980–1.066)	0.005	1.152 (1.043–1.272)
cpRNFL thickness	0.238	0.991 (0.976–1.006)	0.833	NA

All the univariate variables in the table are included in the multivariate analysis.

OR: odds ratio; CI: confidence interval; MD: mean deviation; RNFL: retinal nerve fiber layer; IOP: intraocular pressure.

Variables were further compared between temporal and concentric shape of beta zone in the PACG group and POAG group, respectively. In the PACG group, temporal shape of beta zone eyes showed a significant younger age (*P* = 0.003), mild MD value (*P* = 0.048); while in the POAG group, temporal shape of beta zone eyes showed a significant younger age (*P* = 0.004) and longer axial length (*P* = 0.004).

Since the parapapillary gamma zone may not appear simultaneously with the beta zone, the distribution of beta + gamma zone type versus beta zone only type among concentric/temporal shape of parapapillary beta zone was also analyzed. Among eyes with temporal shape of beta zone (n = 90), 56.5% eyes (26/46) had parapapillary gamma zone in the POAG group, 13.6% eyes (6/44) had parapapillary gamma zone in the PACG group (*P* < 0.001, Table 5). Moreover, among eyes with temporal shape of beta zone, the mean axial length was significantly longer in eyes with beta + gamma zone type than those with beta zone only in the POAG group (25.75 ± 1.23 *vs*. 24.17 ± 0.99 mm, *P* < 0.001), while such difference on axial length was not observed in the PACG group.

**Table 5:** The distribution of beta/gamma zone combination among concentric/temporal shape of parapapillary beta zone.

Beta/gamma combination	Concentric shape (n = 45)		*X*^2^	*P*-value	Temporal shape (n = 90)		*X*^2^	*P*-value
	POAG (n = 23)	POAG (n = 22)			POAG (n = 46)	PACG (n = 44)		
Beta + gamma zones (n, %)	5 (21.7)	6 (27.3)	0.186	0.666	26 (56.5)	6 (13.6)	18.051	< 0.001
Beta zone only (n, %)	18 (78.3)	16 (72.7)			20 (43.5)	38 (86.4)		

PACG: primary angle-closure glaucoma; POAG: primary open angle glaucoma. Chi-square test was performed.

## Discussion

Our results suggest that comparing the PACG and POAG groups that adjusted for age and MD value, the area and shape of OCT-defined parapapillary beta zone exhibited no significant difference, while OCT-defined parapapillary gamma zone was larger in the POAG group. A larger parapapillary beta zone was correlated with older age, severe MD value and longer axial length. Concentric shape of parapapillary beta zone was correlated with older age and worse glaucomatous damage. Moreover, MD value was correlated with the area and shape of parapapillary beta zone in the PACG group, but not in the POAG group. Axial length was correlated with the temporal shape of beta zone in the POAG group, but not in the PACG group.

In both POAG and PACG groups, as well as previously reported in normal eyes^7^, the area of parapapillary beta zone is strongly correlated with age. Curcio et al. has demonstrated that there was an age-related RPE atrophy or degeneration in peripapillary region of healthy donor eyes^12^. It is also evidenced by our previous study that the vessel density of both retina and choroidal microvasculature were decreased in beta zone, which suggested that a microcirculatory deficiency in parapapillary beta zone may exist in normal eyes^13^. It is plausible that choroidal microvascular reduction might accelerate the peripapillary RPE degeneration, which led to the formation and enlargement of aged-related parapapillary beta zone.

One intriguing finding is that the size of OCT-defined beta zone is correlated with the MD value in the whole group, and taken separately in PACG group, but not in POAG group. We then performed a paired sample t-test in eyes with bilateral POAG to further verify this finding. The results also showed that there was no significant difference on beta zone area between severe eyes and mild eyes whose MD value differed by more than 2 dB. In reviewing the literature, conflicting results on relationship between ophthalmoscopic beta zone and visual field MD existed in eyes with POAG. Jonas et al. reported a positive correlation between beta zone area and visual field MD^14^. Jung et al. reported recently that beta zone area was not associated with visual field MD^15^. To the best of our knowledge, there is no report on the direct relationship between OCT-defined parapapillary (new) beta zone and visual field MD in either glaucoma type.

Aside from investigating the size of beta zone, we further divided the shape of parapapillary beta zone into temporal and concentric groups in order to better understand its clinical characteristics. The angular extent of ophthalmoscopic beta zone has been previously addressed^16,17^. Song et al. reported that concentric-type parapapillary atrophy is usually observed in older POAG patients with relatively short axial length^16^. By differentiating parapapillary gamma zone from ophthalmoscopic beta zone, our data showed that concentric shape of beta zone were correlated with older age and worse glaucomatous visual field defect when compared to temporal shape of beta zone in the whole study group. Moreover, the shape of beta zone was correlated with MD value in PACG group, but not in POAG group. Therefore, both the area and shape of parapapillary beta zone showed a closer relationship to visual field MD in eyes with PACG than those with POAG in the current study.

The above findings could be better explained if we assume that the development of the parapapillary beta zone may not share same etiology. In addition to the factors of aging and glaucoma, it has been reported that parapapillary beta zone was correlated with increasing axial length in normal and POAG eyes, although in a lower degree when compared to parapapillary gamma zone^5,7^. Moreover, the current data showed that the temporal shape of beta zone was positively correlated with axial length in the POAG group, but not in the PACG group. Of note, among temporal shape group, the proportion of beta + gamma zone type was significantly higher than that of beta zone only type in POAG group. And the axial length was significantly longer in eyes with beta + gamma zone type than those with beta zone only in POAG group. Taken together, these data indicated that myopic axial elongation may play a role in the development of temporal shape of parapapillary beta zone in POAG eyes.

Several lines of evidences have revealed clues about the mechanism on the development of the parapapillary beta zone. A recent Boramae Myopia Study discovered that both the width of temporal shape of gamma zone and beta zone may increase during axial elongation in healthy children, suggesting mechanical stretching may increase the beta zone during eyeball growth^18^. On the other hand, Wang et al. reported that through dark room provocative tests, some eyes with IOP elevation showed a folding and sliding of the RPE at the end of the peripapillary Bruch’s membrane^19^. One may speculate that other than senile changes, either IOP elevation or myopic axial elongation may exert biomechanical strains on the peripapillary region^20,21^, which may gradually induce the degeneration of parapapillary RPE and lend to the formation of parapapillary beta zone. In the present study, since the eyes with PACG is much less myopic than POAG eyes, it is plausible that the development of parapapillary beta zone in PACG group may mainly induced by aging and glaucoma related biomechanical strains, in accordance with our findings that the parapapillary beta zone showed a correlation with visual field defect MD in eyes with PACG, while those with POAG did not.

As for parapapillary gamma zone, our study showed that the gamma zone area is much larger in POAG group than that in PACG group that matched for age and visual field MD. These results are in accordance with the multivariate analysis that a larger gamma zone is only associated with longer axial length, but not correlates with the glaucoma type. Our data also showed that the area of gamma zone was not significantly correlated with the area of the beta zone, suggesting that both zones were not directly depending on each other in both glaucoma types. Boramae Myopia Study has indicated that the development of gamma zone may be due to changes of border tissue from vertical or internally oblique to externally oblique during axial elongation in myopic children eyes^22,23^. One may speculate that parapapillary gamma zone may be detrimental to the glaucomatous optic neuropathy, since myopia is a recognized risk factor for POAG. However, it has been reported that a larger gamma zone was correlated with slower glaucomatous progression, indicating a potential protective role of gamma zone^24^. Future longitudinal studies are warranted to explore the change pattern of border tissue and anterior sclera canal with different shape involved in the onset and progression of glaucomatous optic neuropathy.

There are several limitations to this study. First, the mean axial length in POAG group is significantly longer than that in PACG group. The limited sample size after further matching for axial length in both groups influenced the testing power of statistical significance. However, since hyperopia and myopia are risk factors for PACG and POAG respectively, our data cohort was representative of a typical patient sample in glaucoma clinics. Second, all of the patients were Chinese. The present findings might not pertain to other ethnic groups. Third, this study was a cross-sectional analysis so that we could not observe the development of parapapillary beta and gamma zones in the course of glaucoma, which may shed light on the mechanism on beta zone formation. Longitudinal studies are warranted to investigate the peripapillary microstructural change in glaucoma and axial myopia.

In conclusion, the current study demonstrated that OCT-defined parapapillary beta and gamma zones exhibited different characteristics in two types of glaucoma. Aside from the effect of aging and glaucoma, myopic axial elongation may play a role during the development of parapapillary beta zone in POAG. Clinically, the size of parapapillary beta zone may serve as a better indicator for glaucoma severity in eyes with PACG than for eyes with POAG.

## Subjects and methods

### Subjects

The study population consisted of glaucoma patients from Shanghai Eye & ENT Hospital of Fudan University from June 2017 to November 2019. The study protocol was in accordance with the tenets of the Declaration of Helsinki for research involving human subjects and approved by the Ethics Committee of the Eye & ENT Hospital of Fudan University. All study participants consented to the examinations before they were performed.

The inclusion criteria were glaucomatous optic nerve head changes with corresponding reliable visual field defects for both POAG and PACG patients. Patients with secondary glaucoma, a combined mechanism for glaucomatous optic neuropathy, history of intraocular surgery, and retinal pathologies including diabetic retinopathy and hypertensive retinopathy, nonglaucomatous optic neuropathy, uveitis, ocular trauma, systematic diseases affecting visual field were excluded. The POAG patients were enrolled with open angle under gonioscopy. The chronic form of PACG patients were enrolled if they met the following criteria: (1) a history of increased IOP (> 22 mmHg) on at least 2 occasions with or without glaucoma treatment; (2) iridotrabecular contact no less than 180 degrees on gonioscopy without compression, and a synechial closure of the anterior chamber angle no less than 90 degrees upon indentation gonioscopy; (3) no history and no ocular signs of an acute attack including the appearance of the iris and lens; (4) no combined-mechanism glaucoma. When both eyes met the criteria, only one eye from each patient was randomly selected for statistical analysis. All participants underwent a comprehensive ophthalmologic examination as described previously^25^.

### Assessment of parapapillary (new) beta zone and gamma zone

The Spectralis OCT system was used to scan the optic disc region as described in detail previously^5^. The parapapillary gamma zone was defined as the parapapillary region free of Bruch’s membrane and the parapapillary (new) beta zone was defined as the parapapillary region with Bruch’s membrane and absence of retinal pigment epithelium on OCT images. The area and angular extent of beta and gamma zones was calculated using the tools built into the Spectralis software. The delineation and the measurement of area were performed by two independent examiners (KS and DZ) who were masked to other ocular characteristics of the participants.

Moreover, according to the different location and angular extent of parapapillary (new) beta zone, eyes with beta zone were further classified in the temporal shape and the concentric shape. When the parapapillary (new) beta zone occupied mainly temporal side around the optic disc and less than 270°, the eyes were classified in the temporal shape. When the parapapillary (new) beta zone occupied more than 270° around the optic disc, the eyes were classified in the concentric shape. Of note, parapapillary (new) beta zone may appear only inferiorly or nasally in rare cases, but we don’t have such cases in this cohort.

### Statistical analysis

The propensity score matching was performed by using the “MatchIt” package in R software (version 4.0.1). A 1:1 matched analysis was conducted to adjust age and MD value using the nearest neighbor method. All other statistical analyses were performed using SPSS software 22.0 (IBM-SPSS, Inc., Chicago, IL, USA). One-way analysis of variance (ANOVA) and paired t test was used to compare continuous parameters accordingly. Linear regression was performed to analyze parameters that related to continuous variable (gamma zone area and beta zone area), while logistic regression was used to determine parameters associated with concentric or temporal shape of beta zone. Those parameters which showed significant associations in the univariate regression or with clinical significance were included in a multivariate regression to determine the independent predictors. All *P*-values were two-sided and the statistical significance level was set at 0.05.

## Data Availability

The datasets generated during and/or analysed during the current study are available from the corresponding author on reasonable request.
